# Hospital and Institutional News

**Published:** 1912-12-07

**Authors:** 


### HOSPITAL AND INSTITUTIONAL NEWS.

### "THE HOSPITAL'S" CHRISTMAS SUPPLEMENT.

Our next issue, as usual at this time of year, will be supplemented by a Christmas Appeal Number. This Supplement, which will be sold separately at the price of one penny, forms an expert guide to the needs of the London hospitals, which are classified in such a way as to make their distinctive claims most easily recognisable. In addition to this the Christmas Supplement will contain two special articles dealing with the spirit and inspiration of the work, which is summarised by facts and figures in the separate paragraphs which are allotted to every institution. At Christmas time, which still excites the warmth of heart to which Dickens gave such jolly expression, the fountains of charity in thought and deed are particularly flowing. Thanks largely to the record which The Hospital has year by year been able to give of the way in which Christmas is kept in the hospitals, the lay public has really begun to grasp how much is done by hospitals' staffs to make the patients cheerful and happy at this national festival. But it is important, as nothing excites greater gratitude and generosity in the public mind, that the story of hospital Christmas should be retold every year. We therefore follow the precedent that proved so popular twelve months ago, and invite every class of institution, whether general, special, mental, or Poor-Law, to send us an individual and detailed account of its Christmas festivities. So much individual thought and care are given at this season, so many good ideas are put into force, that it is of great importance to have the detailed story told. The lay press has no room for this. We have; and the tale of who inspires, organises, and shares in the Christmas festivities will strengthen the public support by appealing to the admiration and sympathy of those who are deaf to appeals in their general form.

### STILL ANOTHER HOSPITAL REMOVAL.

Last week we had the pleasure of congratulating the governing body of the Westminster Hospital on their well-conceived plan for removing into some less affluent region of the County of London, where hospital accommodation is at present insufficient for local needs. This week it is the turn of the British Lying-in Hospital, Endell Street, to receive encomium and congratulation for a similar scheme. With the approval of the King Edward VII. Hospital Fund and of the Charity Commissioners the governing body of this well-known maternity hospital proposes to amalgamate with the Home for Mothers and Babies, Woolwich, and the joint charity thus created will be carried on in a new building specially to be erected at Woolwich for the purpose. The title of the amalgamated institution is to be "The British Hospital for Mothers and Babies," and it is announced that it will use also as a sub-title "National Training School for District Midwives." This may be taken to indicate that the very important educational work of the Endell Street School, as well as that of the Woolwich Home, will receive every encouragement in the new institution, and we cannot doubt that this aspect of the work of the charity will benefit the whole of London as well as the immediate vicinity of the British Hospital elect.

### THE REASONS FOR AMALGAMATION.

According to the official statement, the authorities of the British Lying-in Hospital have been led to take this important step by the rebuilding and alteration in character of the district around Endell Street, which forms a north and south connecting channel between Long Acre and Broad Street at the upper end of Shaftesbury Avenue. The construction of the latter improvement must in itself have had a great effect on all the neighbouring property, for it replaced a collection of rookeries and slums near which a lying-in hospital was very well placed. There is still a good deal of small property in the Drury Lane area quite close to Endell Street and in Soho, but it is quite true that a large proportion of the *clientèle* of the hospital has emigrated to other districts through rebuildings and improvements. The creation of maternity wards in several of the large general hospitals has further influenced the decision which has been taken. The governors have felt that they could best realise the primary object of the existence of the charity by this policy of removal, and in adopting the statesmanlike course of amalgamation with an existing institution on the outskirts of London they have shown themselves well worthy of their trust. It is for the people of London to see to it that no pecuniary difficulties shall mar the success of the experiment, to which we wish all success.

### SIR JAMES BARR'S PLEA FOR DREADNOUGHTS.

Speaking at the Liverpool Caledonian dinner last Saturday, Sir James Barr, the president of the British Medical Association, said that five millions was to be spent on administering an Act which nobody wanted, and which would cost eventually thirty millions. This sum, Sir James is reported to have added, would pay for fifteen Dreadnoughts, which would save the life of the nation, but he was perfectly sure that the Insurance Act would not save the life of a single individual. That is characteristically plain speaking from an authority who has never minced his language on this question, and it is not a little interesting to note that hatred of an Act of Parliament should have led the president of the doctors' trade union to put forward so frank a plea for naval reinforcements. The merit of this plea is beyond the province of hospital workers, who, however they regard the demand for an invincible armada, will be grateful for the speaker's repeated criticism of the defects of the Insurance Act.

### THE JESSOP HOSPITAL IMPROVEMENT SCHEME.

We are glad to record the additions and improvements which have been decided on at the Jessop Hospital for Women, Sheffield. Dr. D. J. Mackintosh, M.V.O., has been consulted and has recommended that a new laundry should be built; that the sanitation of the hospital should be overhauled, which will involve the building of two new sanitary towers; that the staff should be provided with a new nurses' dining room; bed and sitting rooms for the second house surgeon recently added to the staff; a consulting room for the honorary medical staff and a clinical laboratory; and bedrooms for the nurses, including a sick room; and that a fresh system of ventilation should be supplied to the greater part of the institution. How do these recommendations compare with the faults pointed out in our Report on the hospital, which appeared in The Hospital on July 6, 1912? "The wards, and especially the sanitary offices in the hospital proper, were on the whole less satisfactory" than any inspected of recent years: that was the condition found in September 1911, when the hospital was visited, a year before the Report appeared; the accommodation for the maternity nurses and midwives in the new maternity unit was severely criticised, as was the absence of proper bathing and disinfection accommodation for the visiting nursing staff. At the head of the Report we recorded a pleasure in hearing that the committee were taking steps to put their house in order, steps which have been co-ordinated in Dr. Mackintosh's scheme. It is interesting to see that the reorganisation substantially deals with the points criticised, and that by awakening the committee to the serious need for improvement definite reforms have been planned to do away with the old defects. No course could be better calculated to inspire the citizens of Sheffield with respect for the committee which has faced a difficult problem so frankly, and we urge them to give the hospital the support which it badly needs to carry through the improvement scheme successfully. The sum of £15,000 is asked for, and we cannot think that Sheffield will allow itself to take second place now that Leeds and Chester have shown how an intelligent interest in the hospitals of their cities can express itself by the raising of very large sums of money for similar schemes.

### THE LATEST THING IN PHARMACOLOGY LABORATORIES.

The new pharmacology laboratories were opened by Sir Thomas Barlow on Wednesday. They form an addition to the three-storey building, known as the Physiology Institute, which was built in the South Quadrangle a few years ago. Under the supervising hands of Professor F. M. Simpson, the architect, two large research rooms have been placed on the first floor, and the equipment is such that power can be conveyed to instruments, such as a sphygmograph, in any part of the room. One clock registers the time divisions of the instruments, while water power for a centrifugal table and electricity for power or light are typical of the elaboration of the furniture. The second floor consists of a students' laboratory, lecture room, and a demonstration theatre. The new laboratories will take the place of the temporary quarters in the Birkbeck Building, where the pharmacology classes have been temporarily held.

### A BACTERIOLOGIST APPOINTED FOR LONDON.

The recommendation of the hospitals committee to the Metropolitan Asylums Board that a research bacteriologist be appointed at a salary of £500 a year has been carried. Speaking in support of the proposal, Dr. Bousfield said that they had simply been warehousing infectious disease for years. They had been treating the symptoms and knew nothing about the disease itself. They now asked that machinery might be set up by which the period of the patients' stay in hospital might be considerably shortened. Professor Smith pointed out that they had now been building hospitals for a great many years to meet the incidence of various infectious diseases and there had been no marked reduction in the incidence of disease. The Board, up to the present time, had done absolutely nothing of a serious nature to prevent the incidence of disease in London yet. There was no other authority in the world which had the same field for investigation and research. This proposal, of course, is only the result of a gathering conviction that the fever hospital and the removal of the infectious patient were not the beginning and end of the public authorities' responsibilities in this matter. Unjust things have been said of fever hospitals, to which occasion was given, not generally speaking by hospital defects at all, but by the fact that the hospitals were largely wasted through the absence of a highly-qualified worker to investigate the causes of the diseases they were built to isolate.

### RESEARCH WORK AND THE INSURANCE ACT.

An important proposal in connection with the Insurance Act was discussed on the motion of Sir Clifford Allbutt at a meeting of the General Medical Council last week. Sir Clifford remarked that if the object of the Insurance Act were the prevention of disease, they would have to undertake a crusade against many other diseases besides tuberculosis, such as rheumatic fever and diphtheria. How were they to carry out such a general movement? It was suggested that next year a small committee should be appointed by the Government to go into this question very fully as to the way in which existing institutions or new institutions might be developed for research as well as for assistance to medical men, and linked up with great powers of consultation with those whose studies lay along special lines. The Council should be in a position to speak very strongly to any such Government committee in respect to medical education. The first scheme that might be pressed strongly was to have one great central institute which should be the resort of all medical men as far as he knew both of England and Scotland—a large central bureau, perhaps in London or Edinburgh. The other proposal was to develop those many institutions which already existed—for example, laboratory institutions in large provincial towns, pathological and clinical laboratories along scientific lines. If they decided to look to the development of local institutions they would see what an enormous influence it might have in districts around them. The President, Sir Donald MacAlister, said the committee would be a kind of watching committee, and the proposer's resolution was carried.

### THE DOCKS' NEW AMBULANCE SERVICE.

The Port of London Authority has instituted a complete motor ambulance service at the docks. The Victoria and Albert, London, Surrey Commercial, and West India Docks are served by electric motor ambulances, which are stationed at four points and work on week-days between 6 A.M. and 10 P.M., except that at the West India Dock, which is available at any hour on account of the frequency of night work. Each ambulance is staffed by a qualified attendant who holds the first-aid certificate of the St. John Ambulance Association. Already an average of eighteen minutes has been established for the time taken between the receipt of the telephone call and the arrival of a patient at the hospital. The cost of maintaining the service will be about £4,000 a year.

### MAURITIUS' GIFT TO TROPICAL MEDICINE.

The Governor of Mauritius has intimated to Mr. Austen Chamberlain that £200 has been voted by the Legislative Council as a contribution towards the fund which, as we noticed last week and previously, he is raising for the London School of Tropical Medicine. The fund has now reached £50,000.

### THE MENTAL HOSPITAL CHAPLAIN.

A correspondence has been proceeding in a lay contemporary on the subject of the mental hospital chaplain's duties and stipend, to which a medical man and a cleric have been the chief contributors. The points at issue appear to be whether or not he is adequately paid, and how his responsibilities compare with those of the medical staff. The chaplain, who, like the medical man, made the mistake of writing anonymously, gives an interesting summary of the duties attaching to this office and the chaplain's position at a county mental hospital, which, as it emphasises the reality of labours that are sometimes overlooked, we summarise shortly here. "The chaplain is not a resident; he lives about three miles away, and has to get to and fro the best he can. The asylum covers a wide area, has about 1,700 patients, and a staff not much short of three hundred. Who is to take the place, for them, of the parish clergymen and the Nonconformist ministers? Does not the work rest upon the chaplain? It has been experienced over and over again that the patients, while discrediting statements made by the medical officers and staff, will believe the chaplain. He can to a certain extent alleviate some of their fears. The chaplain's duties seem to be minimised in order to pay him a comparatively small stipend. The chaplain holds two services on Sundays, Holy Communion once a month, and pays four visits a week. He is librarian as well as chaplain. But how long are the visits? On two days a week they are three hours; one day a week it is four and a half hours; one day a week it is six hours. The chaplain has to put in extra visits because he has been unable to cope with the work. The stipend paid to him for being chaplain and librarian comes to about £180 a year, and a month's holiday in the summer."

Thus the writer compares the chaplain's duties with his stipend, adding that the visiting committees ought not, in his opinion, to allow the medical superintendents to control his facilities of work. In so far as these concern the services who of our readers can doubt that he is right?

### A CENTRAL STERILISING DEPARTMENT.

Plans have just been approved by the governors of the Leicester Infirmary for the erection of a central sterilising department. This has been found desirable for several reasons, principally that in summer time the high temperature of the operating theatres, caused by having high-pressure steam in the sterilising room, makes surgical work uncomfortable. The introduction also of dressing boxes in the interests of aseptic methods has shown that the two present sterilisers are not sufficient, inasmuch as dressing boxes cannot be packed in as were the old dressing packets, and the increasing work in the theatres makes additional accommodation a necessity. A site has been chosen in the grounds accessible from all parts of the building, and the cost of building the new department will be £400. The removal and fitting up of the present sterilisers and the purchase of an additional one will make the total cost £600.

### SIR RICKMAN GODLEE AND THE VOLUNTARY SYSTEM.

Last week Sir Rickman Godlee, president of the Royal College of Surgeons, had some interesting remarks to make on the growth and prospects of the cottage hospital. Originally, he recalled, when opening the Epping Cottage Hospital, that the first cottage hospital excited the opposition of medical men, who misunderstood how they as well as the patients would benefit from its erection. To-day there were some six hundred and fifty of these institutions in the United Kingdom. In isolated districts they were absolutely essential. Sir Rickman Godlee went on to say that he thought it even more important that small hospitals should be independent of State or municipal control than large ones. What State and municipal control has done for the German hospitals by turning the patients into clinical material, whose deaths are chiefly regretted because the hospital's recovery average will suffer, is being explained in our articles on the subject. We think State control equally bad for hospitals of all sizes, and cannot see from Sir Rickman Godlee's able plea for the voluntary system why State control should be less evil in larger institutions.

### SHARP PRACTICE AT A CHARITY MATINEE.

There is always a temptation at church bazaars, matinées for benevolent purposes, and charity shows generally to sacrifice everything to a big taking, and from time to time it is necessary for those who desire the best interests of voluntary charitable work to point out the growth of abuses. The matinée on Wednesday last week of the fairy play called "The Triumph," which was performed at the Court Theatre under the auspices of the Women's Imperial Health Association and the Women's National Health Association of Ireland, provides a case in point. A lady in the audience being offered a programme for ten shillings by one of the sellers, who were in nurses' uniform, handed to her a sovereign, and, on asking for the change, was informed that no change was being given. The lady, naturally very much taken aback as no notice was visible announcing this important alteration of practice, explained that she must have change as she had no other coin in her possession, and that she would be unable to get home unless change was supplied. The nurse, for so the sellers were described on the programme, still refused, and, as the lady had not sufficient presence of mind to return her programme, which proceeding would have obliged its vendor to disgorge the coin, we imagine that no redress was given. It may have been, for we did not wait after the performance to see the end of the incident. We had seen quite enough to excite our resentment already. To refuse to give change at any matinée is an indefensible and silly proceeding even when the fact is announced conspicuously. To refuse to give it without such an announcement is a form of sharp practice, all the worse for cloaking itself under the name of charity.

### MR. H. S. WELLCOME'S HISTORICAL EXHIBITION.

For some years past Mr. H. S. Wellcome has been organising an historical exhibition of rare and curious objects relating to medicine and the allied sciences. This he has decided to hold next year in London at the same time as the International Medical Congress in August. Mr. Wellcome's own collection will doubtless form a considerable part of the exhibition, and he is also appealing to all who possess objects of historical medical interest to loan them for the exhibition. We trust that Mr. Wellcome will constitute a special section for hospital exhibits, and that an effort will be made to obtain some of the curious and interesting relics which pretty well every old-established hospital has in its possession, though they are often well nigh forgotten or relegated to some dusty lumber room. As an example of our meaning it will suffice to quote the celebrated couch, preserved in the Board Room of St. George's Hospital, upon which John Hunter died when he was attacked with angina pectoris during a board meeting of the hospital. Many hospitals, too, possess valuable paintings of their early benefactors and medical staff by English masters of the eighteenth century; and practically all have museums from which curios, not pathological or gruesome, could be sent to the exhibition. The half-sovereign which was extracted from the windpipe of Brunel, the railway pioneer, was stolen, we believe, a few years ago from one such museum; but there must be plenty of curios connected with eminent people scattered through the hospital museums of the United Kingdom.

### A SCHOOLBOY ON THE OPERATING THEATRE.

A few weeks ago we recorded the visit of some representative boys from the Council Schools of West Ham to the West Ham and Eastern General Hospital, which the boys assist by contributing to its funds. We also added that Mr. Thomas Alexander Cook, the chairman of the board of management, had offered a prize for the best essay describing their visit. We have been now favoured with a copy, and have read it with great interest. The question that rose to our minds before we began to read was this: "Will the writer seem inspired by those in authority to throw indiscriminate praise over everything?" Of course there was a good deal of that; what young observer could fail to be awed by the sense of organisation and the spirit of efficiency which impresses everyone who enters a big institution for the first time? Here is what the boy thought of the theatre: "The operating table was something like an ordinary stretcher, about 3 feet from the ground. It can be raised, lowered, or moved about in several directions as the case requires. The light, both artificial and natural, is of the very best, as life or death depends on what is done in this room. I was pleased to see that the patients are spared the sight of this room as they are made unconscious in a comfortable room adjoining it." Anæsthetists may smile at the adjective applied to their department, but the genuineness of the writer's interest is apparent throughout, and was shared, we have no doubt, by less skilful writers than he. The essay is proof, in short, that these visits are valuable to hospital and schoolboy, and Mr. Cook has only given the necessary fillip to the visitors' interest by the capital idea of offering a prize.

### BUMBLEDOM IN MODERN LONDON.

On October 26 last we ventured an opinion that the Board of Guardians and the Governors of Guy's Hospital include level-headed men of the world, who have only to meet in friendly discussion to secure the adoption of an adequate remedy for the avoidable sufferings and miseries now imposed upon the sick poor of Southwark. Guy's Hospital, as we have shown over and over again, has maintained a very proper position in regard to the Poor-Law sick cases, which require admission at all sorts of hours. The Southwark Guardians have failed to provide necessary ambulance and hospital accommodation in the heart of the district—that is where the needs of the sick poor of Southwark demand it. It is no part of the duty or work of Guy's Hospital habitually, or at all, to undertake the hospital service which is imposed by law upon the Southwark Guardians, and which they have constantly failed to provide in the very centre where it is urgently needed. We are not surprised, however, that the Southwark Guardians should sweep aside the suggestion of a friendly conference, but we are surprised that they should have had the temerity to advertise their demerits by addressing themselves to the Local Government Board, the King's Hospital Fund, and other bodies. We welcome the Southwark Guardians' action in addressing the Local Government Board, because their request for an inquiry will, we hope, be at once granted by the President, Mr. John Burns, so that the whole of the facts may be brought out in evidence. The assured result must be that the present grievous suffering shall be no longer aggravated by the failure of the Board of Guardians of Southwark Union to fulfil their plain duty by providing a central reception station, with the necessary beds, where the public interest requires it should be placed. There can be no question of money in a matter of such urgency. Now the matter is in the hands of the Local Government Board we cannot credit that Mr. John Burns will longer suffer the present scandal to endure in Southwark, without the institution of an immediate and thorough inquiry. This should be followed by the prompt application of an adequate remedy. It is intolerable that modern London should be longer exhibited to the world, through the default of those responsible, as tolerant of this centre of Bumbledom in its worst and most grievous form.

### THE SECRET OF SUCCESSFUL APPEALING.

It is commonly lamented at annual meetings and by speakers on behalf of hospitals at social gatherings that if only people could understand the work done they would subscribe the necessary money. Prince Alexander of Teck, in speaking of what may be called the technique of appeal propaganda last week, went a step further than this. "I have invariably found," he said, "that when the importance of a hospital's work and the need for its efficient maintenance are fully explained in any quarter practical sympathy is forthcoming." In that undoubtedly lies the secret of successful appeal-making. How to interest people, and to get behind the pious and insincere phrases which have made most annual meetings so dull, that is the question. As in everything else—there is a right way and a wrong way to set about it. One can adopt the meretricious allurements of commercial advertisers and recount or magnify this or that detail of hospital procedure in the hope of compelling public interest by the number of pills consumed or such like irrelevancy. That perhaps is a method more forced upon the great institutions of large cities than elsewhere. There is the alternative, to which Prince Alexander of Teck referred in his speech, of really taking some trouble to inform a suitable section of the public of sufficient facts to give them the interest which comes from understanding and understanding alone. This method has done wonders at Leicester and Leeds, for instance, and every hospital now thinking of appealing for support at Christmas time could do no better than to take another of Prince Alexander's remarks as a guide: "When I fail to produce a generous response I am inclined to attribute my failure not to the dullness of the charitable instincts of the individual, but to my own inability adequately to press the claims of their cause." It is a mistake to regard hospital facts and figures as dull, they are never dull when used as symbols of living work and growth.

### QUEEN ALEXANDRA DAY.

The occasion of Queen Alexandra's birthday on December 1 has been signalised by an official list of distributions to charities—chiefly, of course, hospitals—of funds collected last summer through the sale of artificial flowers in the streets. The long delay has doubtless been unavoidable, but it is regrettable because it probably accounts for some wild rumours which were circulating a little while ago of frauds in connection with the collection. The publication of the audited accounts, which is promised shortly, will finally quell all such ridiculous *canards*. The grand total so far allotted amounts to £10,600, and a small balance is still in hand for further distribution. The largest sum given to any one charity is £500 to the Church Army, whose magnificent work is familiar to all. We do not think any hospital worker will grudge this gift. King's College Hospital gets £350, University College Hospital £300, Westminster Hospital £150, London Hospital £300, Charing Cross Hospital £150, St. Mary's Hospital £200, the Royal Free Hospital £200, and the West London Hospital £150. The remaining teaching hospitals do not appear on the list, which is a notable one, though some very well-known hospitals do not figure in it.

### HOSPITAL ACCOMMODATION FOR THROAT, NOSE, AND EAR DISEASES.

An interesting table has been compiled by the *Journal of Laryngology, Rhinology, and Otology* showing the number of beds in the hospitals of British university towns which are allocated to the service of these specialities. There are, of course, many other beds in provincial hospitals into which such cases are admitted, and even some which are earmarked for this purpose. But, broadly speaking, the university clinics contain the great majority of such specially allocated beds, and for teaching purposes these alone are worth enumerating. London comes very well out of the scrutiny, for it affords accommodation equal to that of Edinburgh, Manchester, Leeds, Sheffield, Newcastle, Liverpool, Birmingham, Bristol, Cardiff, Dundee, and Aberdeen all rolled into one. The Metropolis, indeed, contains 263 beds for ear, nose, and throat cases alone, distributed amongst nineteen hospitals. Manchester would appear to be next best off with fifty-two beds, and then Birmingham with forty-eight. It has to be remembered that in some important hospitals cases within the limits of these specialities are admitted into the general surgical wards as and when necessary; this may account for apparent inconsistencies in the published tables of reserved beds in different towns; and it has also to be kept in view that the vast preponderance of these cases are dealt with in out-patient departments and never enter the wards at all.

### THE DAVID WALDIE CENTENARY.

A correspondent writes that Linlithgow, the native town of Dr. David Waldie, who brought the anæsthetic properties of chloroform to the notice of Sir James Simpson, is considering whether his centenary ought not to be honoured by a memorial to him. A proposal is on foot to place a bronze tablet, with an inscription, on the house in which he lived at the time when he made the discovery.

It may be recalled that it was on November 4, 1847, that Professor Simpson made his first experiment with the preparation which Dr. David Waldie had supplied. Point is given to this by the remark of the Professor's describing what happened in a subsequent letter. "The first night we took it Dr. Duncan, Dr. Keith, and I all tried it simultaneously, and were all under the table in a minute or two." There is no need to enlarge on the possibilities which chloroform as an anæsthetic has opened up, of the comfort and ease with which it allows the surgeon to operate, and of the immense number of operations which it has thus made possible. But there is one obvious fact about anæsthetics which, though hardly anyone has had no experience of them, is generally overlooked—namely, the discomfort amounting to pain which the patient experiences on recovering from their effects. While it is thus true to say that anæsthetics have made modern surgery possible, it is untrue to talk of painless surgery. The pain begins, we admit, after the wound has been dressed, but it exists, and no satisfactory means have been found of combating it. This, however, detracts not from the praise due to Dr. Waldie for his early recognition of the value of chloroform, but from the hasty assumptions of those who are not conversant with post-anæsthetic conditions. His centenary should certainly be honoured in Linlithgow.

### MUSIC ACCOMPANIED BY KNIVES AND FORKS.

Torquay, so far as climate, situation, temperature, and sunshine are concerned, is one of the most attractive places, and has often proved one of the best health-giving centres in the British Islands. Judging by the number of its visitors, it has not attained equal popularity with a place like Bournemouth. The latter town has the advantage of an active and businesslike municipality, which neglects no opportunity to add to its attractions, and to provide everything calculated to win appreciation from its visitors. The municipality of Torquay, stimulated by the example of Bournemouth, has recently spent a large sum of money in erecting, near the sea, a pavilion which combines the twofold attraction of orchestral concerts and a large restaurant. It is hardly credible, but it is true, that this restaurant is only divided from the concert chamber by a relatively low wooden partition. The result is, that the numerous visitors who patronise the excellent music provided, especially in the evenings, find when the restaurant is fairly full that it is impossible to enjoy the music, or even to listen to it in comfort. The orchestral chamber and the restaurant are practically one and the same hall. Could municipal stupidity go farther, seeing that the whole of the existing trouble, at relatively little expense, could be entirely obviated by a complete segregation of the restaurant from the hall in which the concerts take place? We call attention to the facts here given, because the health of visitors to Torquay cannot be promoted whilst their tempers may be permanently injured through having to endure the avoidable miseries here brought to light.

### THE SALARIES OF PUBLIC DISPENSERS.

The Lambeth Board of Guardians have been engaged in considering the salaries of their dispensers, and have already induced the Local Government Board to sanction an increase of £10 a year to Mr. E. Darch, dispenser at Montford House Relief Station. His salary will now be £120 a year, plus a further £60 a year for other duties, whilst the value of house, gas, light, and water are estimated to bring the total emoluments up to £222. On the other hand, the Board has only decided to grant Mr. P. S. Thomas, dispenser at the infirmary, a gratuity of £5 for extra services. The salary attaching to his post is £180 a year, and, in addition to the dispensing of medicine for the infirmary, he has also dispensed for two out-relief districts. Since his appointment he has inaugurated a central drug-store at the infirmary, which, according to Mr. C. H. Baldwin, a member of the Board, has saved the Guardians a large sum of money. A project is on foot for Mr. Thomas to take over the dispensing of medicine for the poor in two more out-relief districts, for which it is suggested he should receive a further £20. When the latter arrangement comes before the Guardians for final ratification there should be a favourable opportunity to reconsider the whole position. The Public Pharmacists' and Dispensers' Association are sending round to Boards of Guardians the reply that they have received from the President of the Local Government Board, that, though rejecting any general revision of the present scale of salaries to dispensers, the Board would be prepared, if the Guardians so desired, to allow a maximum salary of £200 per annum after long service or in exceptional cases.

### THIS WEEK'S DRUG MARKET.

Little or no improvement in business has been noticed during the week, and noteworthy changes in the values of drugs have not been numerous. The price of carbolic acid is barely maintained. Somewhat lower prices are asked for balsam copaiba. Balsam tolu continues to. fall in value. Apiol is dearer. Chiretta is lower in price. Cubebs also tend lower. Epsom salts are dearer. Eucalyptus oil continues to rise in value as a result of shortage in supply. Oil of sandalwood is substantially dearer owing to the very high prices paid for sandalwood in India. Cod-liver oil is neglected. A fairly large business has been done in quinine, but makers have announced no change in their quotations. There is practically no change in the position of opium, but it is thought that if hostilities in the Near East ceased the price of the drug would advance.

### TO OUR READERS.

Contributions are specially invited from any of our readers to these columns. They should deal with topical subjects and news. They must be authenticated for the information of the Editor only. The minimum payment if published is 5s. There is no hard-and-fast rule as to space, but notes of about twenty lines in length are preferred.

